# In vivo assessment of the antischistosomal activity of curcumin loaded nanoparticles versus praziquantel in the treatment of *Schistosoma mansoni*

**DOI:** 10.1038/s41598-020-72901-y

**Published:** 2020-09-25

**Authors:** Kamal El-Din M. Mokbel, Ibrahim R. Baiuomy, Abd El-Hamid A. Sabry, Mona M. Mohammed, Marwa A. El-Dardiry

**Affiliations:** 1 Department of Medical Parasitology, Faculty of Medicine, Fayoum University, Fayoum, Egypt; 2 Department of Immunology and Parasitology, Theodor Bilharz Research Institute, Giza, Egypt; 3 Department of Pathology, Theodor Bilharz Research Institute, Giza, Egypt

**Keywords:** Drug discovery, Diagnostics, Drug delivery

## Abstract

Schistosomiasis is a serious parasitic infection affecting millions worldwide. This study aimed to explore the anti-schistosomal activity of curcumin and curcumin loaded gold-nanoparticles (Cur-GNPs) with or without praziquantel (PZQ). We used six groups of the C57BL/6 mice in which five groups were infected with *Schistosoma Mansoni (S. mansoni)* cercariae and exhibited, separately, to different treatment regimens of curcumin, curcumin loaded nanoparticle, and PZQ, in addition to one untreated group which acts as a control. Mice were sacrificed at the 8th week where both worms and eggs were counted in the hepatic and porto-mesenteric vessels in the liver and intestine, respectively, in addition to a histopathological examination of the liver granuloma. Curcumin caused a significant reduction in the worms and egg count (45.45%) at the 3rd week. A significant schistosomicidal effect of PZQ was found in all groups. Cur-GNPs combined with PZQ 97.4% reduction of worm burden in the 3rd week and the highest reduction in the intestinal and hepatic egg content, as well, besides 70.1% reduction of the granuloma size. The results suggested the curcumin in combination with PZQ as a strong schistosomicidal regimen against *S. mansoni* as it alters the hematological, biochemical, and immunological changes induced.

## Introduction

Schistosomiasis or bilharzia is a serious endemic parasitic disease that is prevalent in many countries worldwide. Schistosomiasis is considered the second most disease induced by the parasitic infection induced by *Schistosoma* after *Malaria* due to its effects on public health which further affects the universal economy. As previously reported, the variable consequences of all known species of *Schistosoma* represent severe life-threatening outcomes that may develop to complicated medical conditions such as colorectal and hepatic cancers^1^. Infection by *Schistosoma mansoni* (*S. mansoni*) is an endemic pathogen in North Africa, the Middle East, and Brazil and often progressed to severe hepatic diseases including liver fibrosis or necrosis^2^.

Praziquantel (PZQ) is an anti-worm medication that was developed in 1977^3^. PZQ is considered the only available treatment used in the controlling of the infections induced by *Schistosoma* species^4,5^. Previous studies showed the limited efficacy of PZQ against schistosomules and immature worms which supports the hypothesis that it’s inadequate for the mass treatment in the high endemic areas^4^. Besides, the regular excessive usage of PZQ results in the rise of resistant strains of *S. mansoni*^6,7^. Multiple failures in preventing reinfection have been reported as well^8–10^. The encouragement of new regimens acting alone, or in combination with PZQ to inhibit the growth of the PZQ-resistant strains by the development of genotoxic and mutagenic changes, safely and effectively, is of global interest^11^. So, the current alert of World Health Organization (WHO) urge researchers to find possible and effective replacements of PZQ in the treatment of schistosomiasis, to overcome its limitations and to increase the medical choices^8–10,12–15^.

In the last decade, great efforts were made to develop new anti-parasitic drugs from natural resources such as plant extracts or phytochemicals^16^. One of these plants is the rhizome of *Curcuma longa* (*C. longa*), “turmeric”, or curcumin (from *Zingiberaceae* family) which is highly regarded as a universal remedy in herbal medicine, with a broad spectrum of pharmacological activities^17^. In industry, curcumin used as a natural flavor and coloring material in several food products such as curry, mustard, and potato chips or even as a natural coloring agent in some cosmetic products^18,19^. In Medicinal research, several in-vitro and in-vivo studies revealed the potent anticancer, anti-viral, anti-oxidant, and anti-inflammatory properties of curcumin^20,21^. Moreover, several recent reports showed that curcumin exerts beneficial effects in animal models of liver toxicity, inflammation and cirrhosis^22^ as well as parasiticidal agents; it was found to be active against *Leishmania*^23–25^ and *Trypanosoma cruzi*^26^. Some studies showed that curcumin induced a significant reduction the levels of Glutathione peroxidase resulting in a strong oxidative stress followed increased Caspase-3 production as an indicator of apoptosis in both sex of *S*. *mansoni*^18^ and *Fasciola gigantica* worms^27^.

Many clinical investigations had confirmed the potential pharmacological benefit and the biosafety of curcumin^28^, the poor solubility or stability in aqueous media or the body fluids‚ high metabolic rate‚ the fast clearance due to limited absorption in gastrointestinal (GI) tract, and decreased bioavailability have limited its usage as a clinical treatment^29^. Previous studies had tried to overcome the obstacles of the clinical application of curcumin by improving its bioavailability, increasing the plasma concentration, enhancing the cellular permeability, and controlling the metabolic processes. one of these approaches is the use of targeting nanoparticles drug delivery regimens which succeeded to provide curcumin with more prolonged circulation and improve cellular permeability, besides the stronger resistance to the metabolic processes of curcumin^30^. Due to these reasons, many studies showed the significant usage of nanoparticles coated with curcumin because of their solubility in water and enhanced activity for different medical application, such as the Curcumin–Loaded Targeted Iron Oxide Nanoparticles^31^ and curcumin-loaded dendritic magnetite nanocarriers^32^ for cancer treatment and the Curcumin-loaded into poly(lactic-co-glycolic)acid (PLGA) nanoparticles for the treatment of *Schistosoma *sp.^33^.

Gold nanoparticles (AuNPs) are commonly used as drug delivery-carriers that targeted the biological cells, in vivo, reduce or control the systemic toxicity, and can be prepared by the homogenization (HPH) method^34^. The Curcumin conjugated AuNPs particles were shown to be, naturally, non-toxic through different in vitro biocompatibility studies, such as the non-toxic effect on the Human colon cancer cell lines, HT-29 and SW-948^35^. Another study reported that the AuNPs of 2–4 nm, 5–7 nm and 20–40 nm are non-toxic to MRC-5 cells^36^. An in vivo Xenograft study showed that the dosage of 20 mg/kg curcumin conjugated AuNPs, twice a week, didn’t cause any significant toxicity to internal organs of the mice^37^. Another in vivo study showed the biosafety of curcumin loaded AuNPs for cancer drug delivery in an MCF-7 Xenograft animal model^38^.

In the current study, we aimed to assess the anti-schistosomal activity resulted from the treatment of curcumin loaded on AuNPs nanoparticles with or without a combination with PZQ through an experimental in vivo application in a biological mouse model. The study was intended to explore the parasitological and histopathological impact of the used remedies in different developing stages.

## Results

### The combination of curcumin and PZQ induced a significant hematological, biochemical and immunological alterations in the infected and treated mice

As shown in Table 1, the infected mice suffered from serious changes in their hematological, biochemical and immunological characteristics. In the infected mice, a significant increase in the levels of SGOT, SGPT, AKP, TNFα, and AFP, where a significant reduction was observed in the levels of TG, Superoxide dismutase, and GSH as compared to the non-infected mice. In the infected treated groups, all of the treatments caused a significant reduction in the SGOT, SGPT, TNFα (*P*-value < 0.001), and AKP (*P*-value < 0.01) levels despite the maximum effect was obtained by the treatment with Cur-GNPs with or without PZQ. None of the treatment could induce a significant reduction in the levels of AFP, despite that the groups of Cur-GNPs with or without PZQ showed the maximum reduction. Despite all the treatments increased extensively the levels of TC, only the treatment with curcumin powder at the 7 days and the group treated Cur-GNPs in combination with PZQ at the 21 days showed almost the same levels as in the non-infected group. Only PZQ could significantly increase the levels of TG to the normal levels (*P*-value < 0.001). The treatment of Cur-GNPs with or without PZQ could significantly increase the levels of SOD (*P*-value < 0.001) and GSH (*P*-value < 0.05).

**Table 1:** The effects of different treatments on the hematological, biochemical or immunological alterations in the *S. mansonai* infected groups.

Experimental groups	Subgroups	SGOT (IU/L)	SGPT (IU/L)	TP (g/dl)	AKP	Globulin (g/dl)	TNFα	AFP	TC (mg/dl)	TG (mg/dl)	SOD	GSH
Non-infected		19.88 ± 1.73	18.13 ± 1.36	6.54 ± 0.12	87.25 ± 4.27	3.6 ± 0.05	15.65 ± 2.11	4.88 ± 1.36	135.13 ± 5.96	108.75 ± 10.69	22.88 ± 1.22	19.64 ± 0.51
1. Infected not treated		74.75 ± 5.42^###^	64.13 ± 5.62^###^	5.21 ± 0.08	113.88 ± 6.98^##^	2.29 ± 0.24	188.13 ± 19.36^###^	14.75 ± 1.75^###^	119.88 ± 10.39	74.75 ± 3.69^##^	8.11 ± 0.93^##^	10.44 ± 0.3^#^
2. Curcumin	Cur-1	49 ± 3.74**	40 ± 5.21**	5.88 ± 0.15	99 ± 1.6	2.74 ± 0.16	66 ± 13.37***	13.88 ± 1.73	132.88 ± 26.49	73.13 ± 4.45	8.31 ± 0.57	11.16 ± 0.58
	Cur-3	46.75 ± 5.26**	46.25 ± 3.62*	5.75 ± 0.09	103.63 ± 2.97	2.76 ± 0.09	102.13 ± 14.17**	14.13 ± 1.46	159.63 ± 7.58***	68.13 ± 6.58	9.06 ± 0.44	10.83 ± 0.27
3. PZQ		51.63 ± 2.72**	47.13 ± 4.22*	6.06 ± 0.09	104 ± 5.48	2.93 ± 0.1	104.63 ± 8.73***	13.38 ± 1.3	170.38 ± 5.24***	114.25 ± 24.1***	11.41 ± 0.64	11.66 ± 0.44
4. Curcumin + PZQ	Cur-1 + PZQ	50.38 ± 2.72**	36.38 ± 2.2***	5.98 ± 0.15	109.13 ± 6.71	3.13 ± 0.09	95.38 ± 14.64***	14.75 ± 1.49	161.63 ± 7.84***	69.75 ± 5.92	8.65 ± 0.45	10.85 ± 0.37
	Cur-3 + PZQ	47.13 ± 3.72**	41.13 ± 2.42**	6.15 ± 0.12	97.63 ± 3.58	2.8 ± 0.09 +	101.75 ± 9.79 ***	15.88 ± 1.96	149.38 ± 14.59**	66.25 ± 1.75	8.69 ± 0.25	11.06 ± 0.48
5. Cur-GNPs	Cur-loaded-1	31.88 ± 1.13***	29.25 ± 1.28***	5.49 ± 0.08	92.5 ± 2.07*	2.9 ± 0.08	91.38 ± 5.68***	11.75 ± 1.04	152.63 ± 10.49**	74.75 ± 3.11	15.96 ± 1.23**	16.08 ± 0.28
	Cur-loaded-3	31.75 ± 1.28***	28.88 ± 1.55***	5.74 ± 0.19	93.38 ± 2.56	2.8 ± 0.08	34.88 ± 3.14***	8.5 ± 1.41	162.25 ± 6.39***	69.38 ± 2.45	19 ± 0.71***	17.28 ± 0.76*
6. Cur-GNPs + PZQ	Cur-loaded-1 + PZQ	31.5 ± 1.2***	28 ± 2.07***	5.66 ± 1.05	92.75 ± 2.82*	3.11 ± 0.08	37 ± 2.73***	9.63 ± 1.06	156.5 ± 10.53***	68.25 ± 3.5	14.74 ± 1.06*	16.86 ± 0.59*
	Cur-loaded-3 + PZQ	31 ± 1.85***	30.5 ± 2.07***	6.31 ± 0.08	93.5 ± 2.51	3.05 ± 0.09	37.38 ± 4.78***	9.38 ± 1.19	130.75 ± 6.76	71.5 ± 4.41	19.54 ± 0.62***	17.48 ± 1.12*

*P < 0.05, **P < 0.01, ***P < 0.001 vs. the Infected not treated mice.

^#^*P* < 0.05, ^##^*P* < 0.01, ^###^*P* < 0.001 vs. the non-infected mice.

### Combined treatment by curcumin and PZQ induced a significant reduction in the worm burden

**Figure 1:**
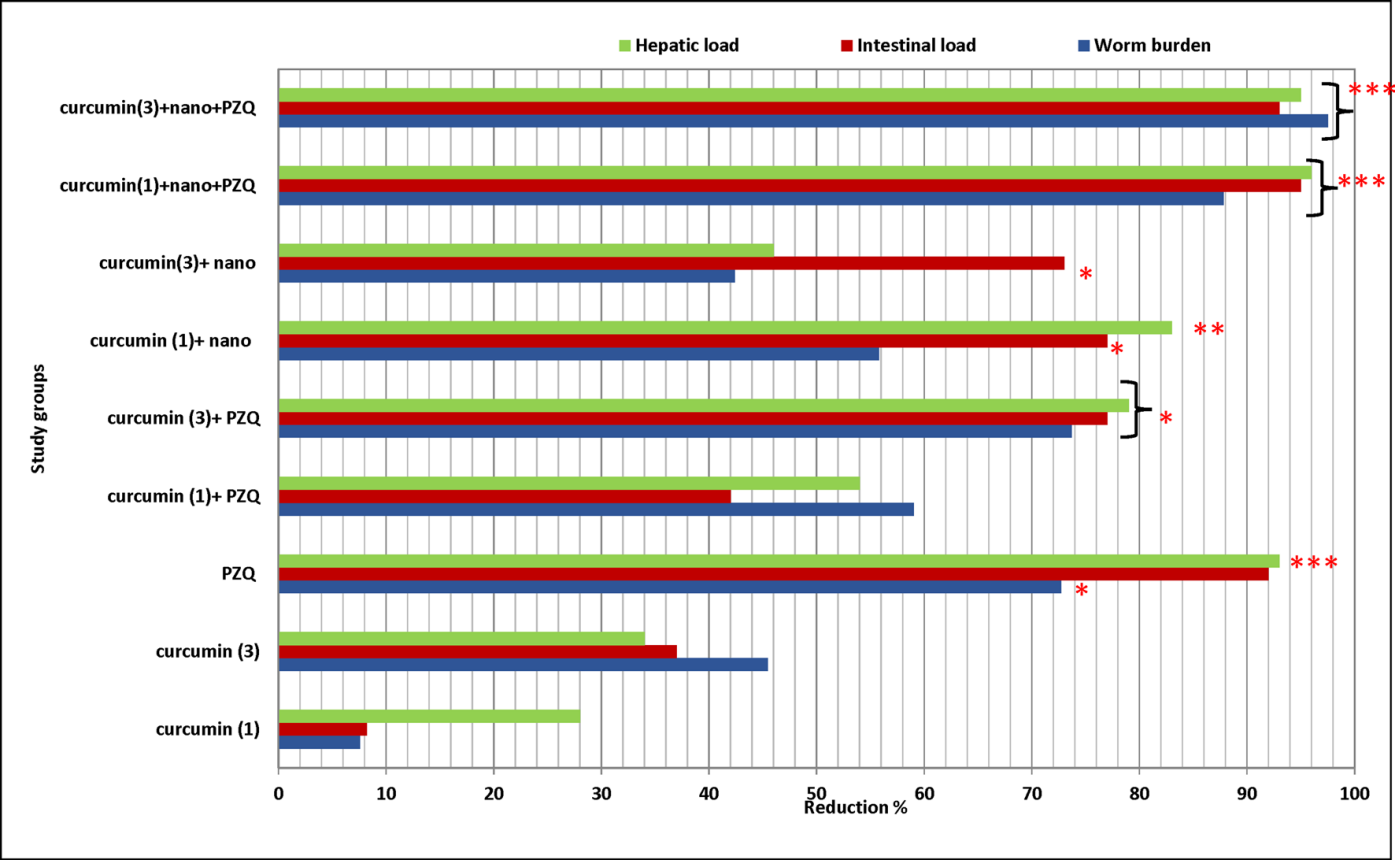
Anti-schistosomal effect of the different treatment regimens. Bar chart summarizes the reduction rate regarding worm burden, intestinal and hepatic load among different experimental groups. The experiments were repeated three times using at least three different samples. *P < 0.05, **P < 0.01, ***P < 0.001 vs. the Infected not treated. The numbers 1 and 3 indicated the 1st and 3rd week of the curcumin treatment, respectively., the symbols, nano and PZQ is a pointed to Cur-GNPs and praziquantel, respectively.

No significant reduction was induced by the curcumin when used as a mono-treatment in the samples taken at the end of the 1st week, compared to the infected non-treated control group, while for the samples from the 3rd week, a significant reduction of 45.45%, was shown (*P*-value < 0.05). PZQ had a significant schistosomicidal effect in all the studied groups, either by mono treatment or combined with curcumin. The maximum reduction of worm burden (97.43%) was obtained in the 6th group which was treated with a mixture of the Cur-GNPs and PZQ at the 3rd weeks pi, followed by the group treated with Cur-GNPs and PZQ at the 1st week pi with a reduction of 87.87%, as shown in Table 2 and Fig. 1. This reduction rate was significantly higher than the results reported in the group treated with PZQ only (*P*-value < 0.001).

**Table 2:** The effects of different treatments on the worm burden in the *S. mansonai* infected groups.

Experimental groups	Subgroups	Detection time (week)	Mean no. of worms	% Reduction
7. Infected not treated		8	33.0	0
Infected treated groups				
8. Curcumin	Cur-1	8	30.5	7.57
	Cur-3	12	18.0	45.45
9. PZQ		8	9.0	72.72*
10. Curcumin + PZQ	Cur-1 + PZQ	8	13.5	59.09
	Cur-3 + PZQ	12	8.7	73.75*
11. Cur-GNPs	Cur-loaded-1	8	14.6	55.87
	Cur-loaded-3	12	19.0	42.42
12. Cur-GNPs + PZQ	Cur-loaded-1 + PZQ	8	4.0	87.87***
	Cur-loaded-3 + PZQ	12	85.0	97.43***

*P < 0.05, ***P < 0.001 vs. the Infected not treated.

### Combined treatment by curcumin and PZQ induced a significant reduction in the egg count and granuloma size

Regarding the intestinal egg count, the infected non-treated group showed significantly higher values than all of the treated groups (*P*-value < 0.001). The highest reduction was achieved in the groups that received curcumin combined with PZQ, either at the first or the third-week pi (95.41% and 93.44%, respectively). Concerning the hepatic egg load, the same groups gave the best results as well (96.80% and 95.46%, respectively), and these results were significant (*P*-value < 0.001) as shown in Table 3.

**Table 3:** The effects of different treatments on the egg load in the *S. mansonai* infected groups.

Experimental groups	Subgroups	Intestinal egg load		Hepatic egg load	
		Mean ± SD	% Reduction	Mean ± SD	% Reduction
1. Infected not treated		38,130 ± 1100	0	46,880 ± 1132	0
Infected treated groups					
2. Curcumin	Cur-1	35,000 ± 378	8.20	33,750 ± 791	28.00
	Cur-3	24,000 ± 586	37.05	30,880 ± 666	34.12
3. PZQ		2675 ± 212	92.98***	3000 ± 167	93.60***
4. Curcumin + PZQ	Cur-1 + PZQ				
	Cur-3 + PZQ	21,750 ± 306	42.95	21,130 ± 223	54.92
5. Cur-GNPs	Cur-loaded-1	8670 ± 116	77.30***	9670 ± 58	79.40***
	Cur-loaded-3	8670 ± 306	77.26***	7570 ± 230	83.85***
6. Cur-GNPs + PZQ	Cur-loaded-1 + PZQ				
	Cur-loaded-3 + PZQ	10,000 ± 100	73.77***	25,000 ± 707	46.67

***P < 0.001 vs. the Infected not treated.

**Figure 2:**
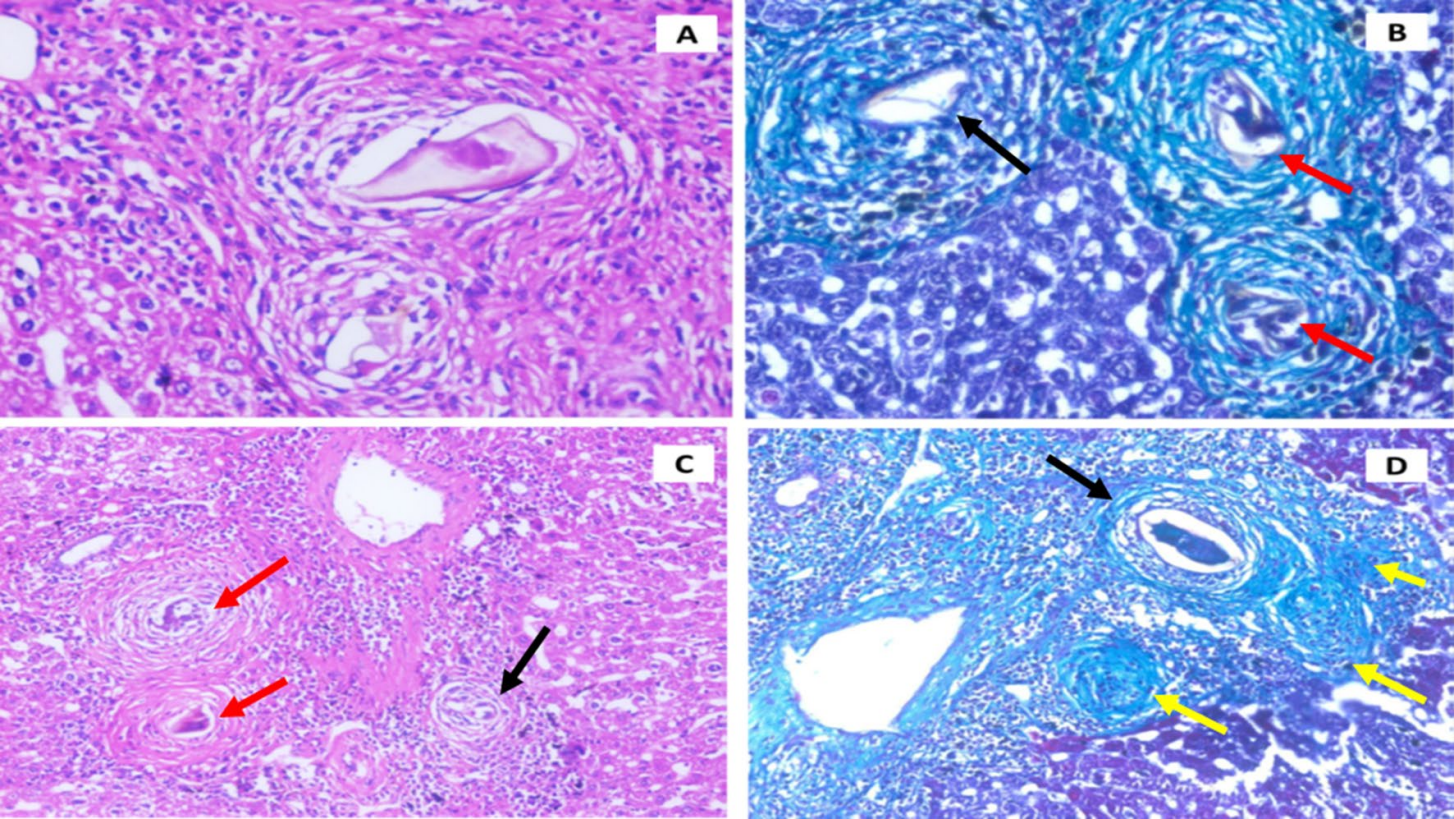
Sections of infected non-treated control mice. Infected non-treated control group shows cellular (black arrow), fibro-cellular (red arrows) and predominantly fibrous (Yellow arrow) granulomas with bilharzia ova. (**A**) Granuloma with bilharzia ova, H&E ×400. (**B**) Granuloma with bilharzia ova, Masson Trichrome ×400. (**C**) Parenchymal granuloma with bilharzia ova. H&E ×200, (**D**) Portal granuloma with bilharzia ova, Masson Trichrome ×200.

In the infected untreated mice, the histopathological screening showed a noticeable rapture of the lobular architecture, where the granuloma, surrounded by ova, were cited in the hepatic parenchyma and the portal tract (Fig. 2). There was a striking reduction in granuloma size achieved with the group that received nanoparticles loaded with curcumin in the 3rd week and PZQ in the 5th week pi with a size reduction of 70.07% when compared to the infected non-treated group and the group that received PZQ only at the 5th week after infection. The normal deposition of granuloma with ova in the infected non-treated mice is shown in Fig. 2.

## Discussion

The main objective of the present work was to study the schistosomicidal effect of mono-treatment of Cur-GNPs with and without PZQ in the mouse model. The effects of curcumin with or without loading to the nanoparticles were studied, as well. The results showed that Curcumin and PZQ induced a significant hematological, biochemical and immunological alterations in the infected mice. In an agreement with these findings, many previous studies showed similar results^39,40^.

The study showed that mono-treatment of *S. mansoni* infected mice with curcumin could not induce any significant reductions in the total worm burdens, the liver egg count, or the intestinal egg count. The total worm burden was non-significantly reduced and didn't exceed 45.45% in the curcumin-treated group. Meanwhile, the effect of curcumin extract alone on tissue egg loads was minor. On the contrary to our results, a previous study reported that the mono-treatment with curcumin might, significantly, reduce both the worm burden and the egg count, but after three months of infection, compared to the results of untreated infected groups^41^. The contradiction might be due to the short treatment duration used in the current study, as 8 weeks instead of three months.

The treatment of *S. mansoni* infected mice with PZQ showed a significant reduction in total worm burden to 72.72%, in intestinal egg count to 92.98%, and in the hepatic egg count to 93.6%, compared to the infected control group. These results are in agreement with previous studies that showed a significant reduction of both the worm burden and egg count in PZQ-treated settings compared to the untreated group^40,41^. On the other hand, we could confirm that the PZQ treatment didn’t show a complete eradication of worms, while most of the male and female worms were remain alive. A previous investigation revealed that at four weeks pi in mice groups treated with PZQ, there were some male worms still alive which agree with our findings^42,43^.

Remarkably, the mono-treatment of Schistosomiasis with curcumin alone was less effective in reducing the worm burden (45.45%) compared to PZQ (72.72%). In the meantime, the combined treatment of curcumin and half-dose PZQ caused a significant reduction in the worm burden (73.75%). More reduction (up to 95.62%) was induced for the intestinal egg count and the hepatic egg count (96.43%). A noticeable worm shift to the liver was recorded which might be a result of retardation of worm normal path in the portal vein. The mono treatment by Cur-GNPs didn’t show any significant reduction of worm burden (42.42%), but the combination with PZQ induces a more significant reduction of worm burden up to 97.49%. In contrast, the intestinal egg load reduction was 73.77%, and hepatic egg load was 46.67% in the mono treatment by Cur-GNPs which increase to 93.44% and 95.46%, respectively when coupled with PZQ. In an agreement with these findings, a previous in vitro study showed that the incubation of *S. mansoni* with different doses of curcumin affected the viability of adult worms and a reduction in the egg count beside it caused a significant separation in worm pairs’^44,45^. Another study demonstrated the down-regulation of two of the Notch receptor genes, the Notch ligand gene, and the co-repressor gene, in *S. mansoni* treated with curcumin which suggests that the mechanism of curcumin inhibitory effect may result from the repression of the Notch pathway in the treated worms^46^.

In the current study, we reported the reduction of the number of eggs per gram of the *S. mansoni* tissue, besides, the abnormal development of ovular stages (oogram pattern) in the intestinal wall of the treated mice which was in an agreement with Aly and colleagues^47^. In another study, a treatment protocol with methanol extract and soluble glycoprotein fraction of *Allium sativum* (*A. sativum*) was applied to target the inhibition of ova production in the hosts exposed to infection by which the further damages of host tissues and organs have been avoided^48^.

In the current work, histopathological investigations of liver sections in the group treated with curcumin only showed a non-significant reduction in granuloma count and diameter compared to the control group. Contrary to our findings, the treatment of *S. mansoni* by curcumin was reported to have an effective reduction of the worm, tissue-egg burdens, and hepatic granuloma^49^. Despite the PZQ was reported to be a highly curative agent, but the insignificant effect on granuloma count and diameters might be considered, where the mice continued to suffer from the disease progression accompanied by the chronic cellular inflammation. Despites it worth to mention that the combined treatment of curcumin and PZQ showed a significant decrease in granuloma count and diameter.

In our study, there was a striking reduction of 70.07% in granuloma size in the group treated with Cur-GNPs at the 3rd week and PZQ at the 5th week followed by the group treated with Cur-GNPs at the 1st week and PZQ at the 5th week with 34.13% reduction in the size compared to either the infected non-treated group or the group treated with PZQ only. Regarding the granuloma’s numbers, the regimen of Cur-GNPs combination with PZQ at the 3rd and the 1st weeks showed the lowest values by 15.22 and 16.40, respectively. In a previous study, it was reported that both methanol extract and soluble glycoprotein fraction of *A. sativum* treatment caused a significant reduction in the number and size of hepatic granuloma among all the treated groups, while the mono-treatment by PZQ caused less reduction^47^.

Our results showed that curcumin induced a slight alteration in the oogram pattern of eggs in the mucosa of the small intestine of the *S. mansoni* infected groups. This caused a non-significant reduction in numbers of intact eggs and a slight increase in numbers of dead eggs as compared to the infected control group, which possesses 95% intact cells and 5% dead cells. These reductions were increased in both mono-treatment with PZQ and in the groups subjected to combined treatment, as well. The reported changes in the count and characteristics of eggs as shown by the oogram will provide a simple, sensitive, and reliable drug-screening tool against *S. mansoni.* It measures the represented effects of the combined treatment regimens on oviposition, maturation, and survival of the eggs trapped in the intestinal mucosa. Therefore, the changes and alterations of the oogram might reflect the efficacy of the combined therapy by interruption of the egg growth and development.

In the current study, the combined therapy regimens exhibited strong ovicidal activity against the ova settled in the intestinal wall. The combined treatment of Cur-GNPs and PZQ caused an efficient eradication of worms with a significant decline in the number of intact mature eggs combined with an expected increase in the number of dead eggs, besides, the reduction in the granuloma number and diameter. These changes were accompanied by the disappearance of active cellular granuloma and an increase in healed fibrous granuloma with the return to the normal architecture of the hepatic strands and lobular structure. In agreement with our results, previous studies showed a reduction in the number of mature ova, an increase in the dead ova counts, and disappearance of immature eggs in mice infected with *S. mansoni* and treated with PZQ while the treatment by curcumin was more effective in the controlling of the disease severity, due to the toxins released by the eggs^40,50^.

Finally, we concluded that curcumin is a remarkable non-toxic plant with many medical properties, but its efficacy as an anti-schistosomal drug is not that much as it might only improve the pathogenic changes induced in *S. mansoni* infected mice. However, the successful combination with PZQ induced significant effects that suggested promising regimens in the treatment of the *S. mansoni* infections.

## Materials and methods

### Ethics statement

All animal experiments were performed under the guidelines of the Egyptian *Institutional Animal Care and Use Committee* (IACUC) https://www.aalas.org/iacuc. This study was conducted following the ethical guidelines of the Theodor Bilharz Research Institute (TBRI), Giza, Egypt (approval no. TBRI 28/21/2016).

### Mice

In the current study, we used 96 female mice of the C57BL/6 strain, for 8 weeks of experiments, that were supplied by the Schistosome Biological Supply Program (SBSP), TBRI, Giza, Egypt. Mice weights ranged from 18 to 20 g and ages of 6–8 weeks old. Mice were housed in polycarbonate boxes, maximum six per box, with steel-wire tops and bedded with wood shavings. The temperature was maintained at 22 ± 3 °C with a relative humidity of 50 ± 15% and a 12-h light/dark photoperiod with free access to food, Purina chow pellets (20% protein), and tap water, at least for 1 week before experiment for the acclimatization. The treatments and surgical procedures were in accordance with the ethical guidelines of TBRI. At the end of the experiment, in the 8th week, mice were anesthetized using inhalant anesthesia of glycol-diluted 30% isoflurane (Sigma-Aldrich, St. Louis, Missouri, USA), then euthanized by cervical dislocation for further experiments. The disposal of the animal carcass was in accordance with the standard operating procedures.

### *Schistosoma mansoni* cercariae strains

*Schistosoma mansoni* cercariae (Egyptian strain) were obtained from infected intermediate host snails (Biomphalaria Alexandrina) and maintained at the Schistosome Biological Supply Center. The infection of mice included in the experimental study took place by the subcutaneous shed of fresh cercariae, 120 ± 10/mouse, as previously described^51^.

### Preparation of curcumin nano-emulsion

The ready-to-use crude powder of *C. longa rhizome* was purchased from Sigma-Aldrich (Sigma-Aldrich, Saint Louis, MO, USA) and the total purity was > 94% as determined by the High Protein Liquid Chromatography (HPLC) and stored at − 20 °C until usage. The curcumin nano-emulsion was prepared from the ethanol extract of crude powder of *C. longa rhizome.* Briefly, 200 g crude powder of the *C. longa rhizome* was mixed with 800 ml of 98% ethanol by vigorous vortexing. The liquid phase was air-dried at room temperature in Petri dishes and the resultant powder was stored in a sterile glass bottle at room temperature^44^.

### Synthesis of Cur-GNPs

The synthesis of Cur-GNPs was performed as previously described by *Shaabani* and colleagues, 2017^52^. Briefly, the curcumin powder, from the previous step, was prepared as 350 µM solution in 7.5 ml distilled water and adjusted to a pH of 9–10 by 150 mM Potassium Carbonate (K2CO3), (Sigma-Aldrich, St. Louis, Missouri, USA). 2.5 ml of the HAuCl4 solution, 4 mM, was added, dropwise to the above curcumin solution and vortexed, vigorously, for 4 h. Rection continued for the next 72 h and the resulted solution was used for further experiments. the characterization of the synthesized Cur-GNPs was estimated by the UV–Vis spectrometer Shimadzu UV-1800 (Shimadzu Schweiz GmbH, Reinach BL, Switzerland) where the absorption of Cur-GNPs was characterized at 517 nm by sharp peaks to indicate the spherical Cur-GNPs that were stable for the next few days. The purity of the produced nanoparticles was estimated by HPLC.

### Experimental design

This study was conducted between February 2017 and April 2017 using six groups of the C57BL/6 mice (16 each). Five groups were infected with *S. mansoni* cercariae while the 6th group was left uninfected to act as a negative control.

The 1st group (curcumin-treated mice) was subdivided into two subgroups (eight each), the Cur-1, treated at the end of the 1st week post-infection (pi) and the Cur-3, treated at the end of the 3rd weak pi. In both of the two sub-groups’ curcumin was injected intraperitoneally at the dose of 400 mg/kg of the body weight, twice/week for eight consecutive weeks, using a stainless-steel oral cannula, as previously described by El-Ansary and colleagues^41^, and Larasati and Colleagues^53^, with little modifications.

In the 2nd group (PZQ-treated group), at the 5th-week pi, a fresh suspension of PZQ (Alexandria Company for Pharmaceuticals and Chemical Industries, Alex., Egypt) was prepared in 13 ml of 2% cremophore-EL (Sigma-Aldrich, St. Louis, Missouri, USA). The mixture was, orally, administered to mice by a dose of (500 mg/kg) for two consecutive days, as described everywhere^54,55^.

The 3rd group (Cur-GNPs group) was further subdivided into two subgroups, Cur-1 loaded + PZQ and Cur-3 loaded + PZQ, similar to the 1st group. A suspension of Cur-GNPs was prepared in a vehicle of 7% (v/v) Tween-80 and 3% (v/v) ethanol, and supplied orally to the animals.

The 4th group (Cur/PZQ-treated group) had a mixed treatment of curcumin and PZQ, at the same doses and timeline, as had been described in the 1st and 2nd groups.

The 5th group (Cur-GNPs /PZQ-treated group) had a mixed treatment of Cur-GNPs and PZQ, at the same doses and timeline, as had been described in the 2nd and 3rd groups.

### Determination of the hematological, biochemical or immunological characteristics

Different biomarkers had been used to study the effect of different treatments on the studied mice groups. At the sacrifice day, blood was obtained from mice by heart puncture where 4–5 ml were obtained from each mouse that have been used in different lab tests according to the manufacturer instructions. The lab tests included the determination of Serum Glutamic-Oxaloacetic Transaminase (SGOT), Serum Glutamic-Pyruvic Transaminase (SGPT), total protein (TP), Alkaline Phosphatase (AKP), Globulin, Tumor Necrosis Factor alpha (TNFα), Alpha-fetoprotein (AFP), Total Cholesterol (TC), Triglycerides (TG), Superoxide dismutase (SOD), and Reduced Glutathione (GSH).

### Study of parasitological criteria

At the end of the 8th week, all the Mice groups were sacrificed by cervical decapitation, followed by the perfusion of the hepatic and Porto-mesenteric vessels which enable the counting of all presented worms^56^.

A. *Worm burden* Adult schistosomes’ recovery was assessed by the Porto-mesenteric perfusion technique, two weeks post-treatment (pt), according to the method of Duvall and DeWitt, 1967^57^. Further, a microscopic investigation was performed to calculate the total number of worms, either single separated male and female worms or copula using a binocular microscope.
B. *Egg count* The absolute count of deposited eggs in the liver and intestine of the studied animal was calculated. Briefly, tiny pieces of the isolated liver tissues and small intestine were collected and frozen at − 20 °C, then the tissues were digested with 4% potassium hydroxide (KOH) and the total number of liver and intestinal eggs was determined by microscopy^58^. The ovular counting at the various developmental stages of each mouse and the mean values were determined, where the measurements were performed in triplicates^59^.

### Histopathological examination

Immediately after sacrifice, the liver organs from each mouse were isolated and divided into two parts; the first part was stored at − 80 °C for biochemical studies whereas the second part was suspended in 10% formal saline and was introduced to the fixation step. Tissue samples were fixed in a solution of neutral buffered formalin (10%), dehydrated, and then waxed by paraffin for micro-sectioning. A total of five sections were prepared from each liver specimen at the sizes of at least 250 μm and a thickness of four microns^60^. Later, these sections were de-paraffinized and stained with H & E stains (hematoxylin and eosin), as mentioned elsewhere^61^.

For a demonstration of the collagen fibers for granuloma counting, the Masson trichrome stains were used where the lesions containing a central single ovum were selected^62^. Briefly, the maximum diameter of the lesion was detected, then the ocular micrometer was rotated by 90 degrees, the perpendicular diameter was measured, and the mean diameter was calculated^60^. The sizes of all liver granuloma were calculated, accordingly, by the mean diameter of each lesion, assuming their spherical shape^62^. The mean count of liver granulomas, from each section, was estimated in five random successive fields with the dimensions of 10 × 10 µm. Reductions in mean counts and diameters of hepatic granuloma in the treated groups were determined and compared to those in the infected untreated control groups. The images were taken by Dr. Kamal El-Din Mokbel, in Theodor Bilharz Research Institute (TBRI), Giza, Egypt.

### Statistical analysis

The data were recorded on an “Investigation report form” which were further uploaded to and analyzed by the statistical software, SPSS version 16 (Chicago, IL, USA). ANOVA and Kruskal–Wallis tests were used to compare the numerical parametric and nonparametric data, respectively. The student’s t-test and the Mann–Whitney-U test were used to measure the violation and homogeneity of the parametric data for all groups. The estimated statistically significant levels were considered at *P*-value < 0.05 or *P*-value < 0.0001 for highly significant results.

## References

[1] Li G (2019). Derivation and external validation of a model to predict 2-year mortality risk of patients with advanced schistosomiasis after discharge. EBioMedicine..

[2] Elbaz T, Esmat G (2013). Hepatic and intestinal schistosomiasis: Review. J. Adv. Res..

[3] Seubert J, Pohlke R, Loebich F (1977). Synthesis and properties of praziquantel, a novel broad spectrum anthelmintic with excellent activity against schistosomes and cestodes. Experientia.

[4] Doenhoff MJ, Cioli D, Utzinger J (2008). Praziquantel: Mechanisms of action, resistance and new derivatives for schistosomiasis. Curr. Opin. Infect. Dis..

[5] Vale N, Gouveia MJ, Rinaldi G, Brindley PJ, Gärtner F, Correia da Costa JM (2017). Praziquantel for schistosomiasis: Single-drug metabolism revisited, mode of action, and resistance. Antimicrob. Agents Chemother..

[6] Melman SD (2009). Reduced susceptibility to praziquantel among naturally occurring Kenyan isolates of *Schistosoma mansoni*. PLoS Negl. Trop. Dis..

[7] Zhang SM, Coultas KA (2013). Identification of plumbagin and sanguinarine as effective chemotherapeutic agents for treatment of schistosomiasis. Int. J. Parasitol. Drug..

[8] Fallon PG, Doenhoff MJ (1994). Drug-resistant schistosomiasis: Resistance to praziquantel and oxamniquine induced in *Schistosoma mansoni* in mice is drug specific. Am. J. Trop. Med. Hyg..

[9] Ismail M (1999). Resistance to praziquantel: Direct evidence from *Schistosoma mansoni* isolated from Egyptian villagers. Am. J. Trop. Med. Hyg..

[10] Pica-Mattoccia L, Cioli D (2004). Sex- and stage-related sensitivity of *Schistosoma mansoni* to in vivo and in vitro praziquantel treatment. Int. J. Parasitol..

[11] El Ridi R, Tallima H, Dalton JP, Donnelly S (2014). Induction of protective immune responses against schistosomiasis using functionally active cysteine peptidases. Front. Genet..

[12] Cioli D, Pica-Mattoccia L, Basso A, Guidi A (2014). Schistosomiasis control: Praziquantel forever?. Mol. Biochem. Parasitol..

[13] Yepes E, Varela-M RE, López-Abán J, Dakir EL, Mollinedo FMA (2014). In vitro and in vivo anti-schistosomal activity of the alkylphospholipid analoge delfosine. PLoS ONE.

[14] Caffrey CR (2015). Schistosomiasis and its treatment. Future Med. Chem..

[15] World Health Organization. Schistosomiasis Geneva, Switzerland 2016 [updated February 2016; cited 2016 October]. Fact sheet N°115]. https://www.who.int/mediacentre/factsheets/fs115/en/

[16] Abou El Dahab MM, Shahat SM, Mahmoud SSM, Mahana NA (2019). In vitro effect of curcumin on *Schistosoma* species viability, tegument ultrastructure and egg hatchability. Exp. Parasitol..

[17] Nasri H (2014). Turmeric: A spice with multifunctional medicinal properties. J. Herb. Med. Pharmacol..

[18] de Paula Aguiar D (2016). Curcumin generates oxidative stress and induces apoptosis in adult *Schistosoma mansoni* worms. PLoS ONE.

[19] Joe B, Vijaykumar M, Lokesh BR (2004). Biological properties of curcumin-cellular and molecular mechanisms of action. Crit. Rev. Food Sci. Nutr..

[20] Aggarwal BB, Harikumar KB (2009). Potential therapeutic effects of curcumin, the anti-inflammatory agent, against neurodegenerative, cardiovascular, pulmonary, metabolic, autoimmune and neoplastic diseases. Int. J. Biochem. Cell Biol..

[21] Tu CT, Han B, Liu HC, Zhang SC (2011). Curcumin protects mice against concanavalin A-induced hepatitis by inhibiting intrahepatic intercellular adhesion molecule-1 (ICAM-1) and CXCL10 expression. Mol. Cell. Biochem..

[22] Lee HY (2017). Curcumin and *Curcuma longa* L. extract ameliorate lipid accumulation through the regulation of the endoplasmic reticulum redox and ER stress. Sci. Rep..

[23] Koide T, Nose M, Ogihara Y, Yabu Y, Ohta N (2002). Leishmanicidal effect of curcumin in vitro. Biol. Pharm. Bull..

[24] Das R, Roy A, Dutta N, Majumder HK (2008). Reactive oxygen species and imbalance of calcium homeostasis contributes to curcumin induced programmed cell death in *Leishmania donovani*. Apoptosis.

[25] Pérez-Arriaga L (2006). Cytotoxic effect of curcumin on *Giardia lamblia* trophozoites. Acta. Trop..

[26] Nagajyothi F, Zhao D, Weiss LM, Tanowitz HB (2012). Curcumin treatment provides protection against *Trypanosoma cruzi* infection. Parasitol. Res..

[27] Rehman A (2020). Generation of oxidative stress and induction of apoptotic like events in curcumin and thymoquinone treated adult *Fasciola gigantica* worms. Exp. Parasitol..

[28] Dende C (2017). Nanocurcumin is superior to native curcumin in preventing degenerative changes in experimental cerebral malaria. Sci. Rep..

[29] Anand P, Kunnumakkara AB, Newman RA, Aggarwal BB (2007). Bioavailability of curcumin: Problems and promises. Mol. Pharm..

[30] Ghalandarlaki N, Alizadeh AM, Ashkani-Esfahani S (2014). Nanotechnology-applied curcumin for different diseases therapy. BioMed Res. Int..

[31] Heydari H, Zarrabi A, Zarepour A (2017). Design and construction of curcumin—Loaded targeted iron oxide nanoparticles for cancer treatment. J. Babol Univ. Med. Sci..

[32] Montazerabadi A (2019). Folate-modified and curcumin-loaded dendritic magnetite nanocarriers for the targeted thermo-chemotherapy of cancer cells. Artif. Cells Nanomed. Biotechnol..

[33] Busari ZA (2017). Antiplasmodial activity and toxicological assessment of curcumin PLGA-encapsulated nanoparticles. Front. Pharmacol..

[34] Gu YJ (2009). Nuclear penetration of surface functionalized gold nanoparticles. Toxicol. Appl. Pharmacol..

[35] Al-Ani LA (2019). Hybrid nanocomposite curcumin-capped gold nanoparticle-reduced graphene oxide: Anti-oxidant potency and selective cancer cytotoxicity. PLoS ONE.

[36] Yen HJ, Hsu SH, Tsai CL (2009). Cytotoxicity and immunological response of gold and silver nanoparticles of different sizes. Small..

[37] Khandelwal P, Alam A, Choksi A, Chattopadhyay S, Poddar P (2018). Retention of anticancer activity of curcumin after conjugation with fluorescent gold quantum clusters: An in vitro and in vivo xenograft study. ACS Omega.

[38] Rao KM, Kumar A, Suneetha M, Han SS (2018). pH and near-infrared active; chitosan-coated halloysite nanotubes loaded with curcumin-Au hybrid nanoparticles for cancer drug delivery. Int. J. Biol. Macromol..

[39] Jiang Z, Wan Y, Li P, Xue Y, Cui W, Chen Q, Chen J, Wang F, Mao D (2019). Effect of curcumin supplement in summer diet on blood metabolites, antioxidant status, immune response, and testicular gene expression in Hu sheep. Animals.

[40] Mahmoud EA, Elbessoumy AA (2013). Effect of curcumin on hematological, biochemical and antioxidants parameters in *Schistosoma mansoni* infected mice. Int. J. Sci..

[41] El-Ansary AK, Ahmed SA, Aly SA (2007). Antischistosomal and liver protective effects of *Curcuma longa* extract in *Schistosoma mansoni* infected mice. Indian. J. Exp. Biol..

[42] Melkus MW (2020). Elucidation of cellular responses in non-human primates with chronic schistosomiasis followed by praziquantel treatment. Front. Cell Infect. Microbiol..

[43] Yang L (2009). Enhancement the oral bioavailability of praziquantel by incorporation into solid lipid nanoparticles. Pharmazie.

[44] Magalhães LG (2009). In vitro schistosomicidal activity of curcumin against *Schistosoma mansoni* adult worms. Parasitol. Res..

[45] El-Kott AF, Mohammed RT, Ismail NR (2011). Efficacy of garlic and mirazid in treatment of the liver granuloma in mice infected with *Schistosoma mansoni*. Res. J. Parasitol..

[46] Morais ER (2013). Effects of curcumin on the parasite *Schistosoma mansoni*: A transcriptomic Approach. Mol. Biochem. Parasit..

[47] Aly I (2017). Efficacy of soluble glycoprotein fraction from *Allium sativum* purified by size exclusion chromatography on murine *Schistosomiasis mansoni*. Microbial Pathog..

[48] Kamel ROA, El-Shinnawy NA (2015). Immunomodulatory effect of garlic oil extract on *Schistosoma mansoni* infected mice. Asian Pac. J. Trop. Med..

[49] Allam G (2009). Immunomodulatory effects of curcumin treatment on murine *Schistosomiasis mansoni*. Immunobiology.

[50] Chou DK, Krishnamurthy R, Randolph TW, Carpenter JF, Manning MC (2005). Effects of Tween 20 and Tween 80 on the stability of Albutropin during agitation. J. Pharm. Sci..

[51] Liang, Y.S., John, B.I., & Boyd, D.A. Laboratory cultivation of schistosome vector snails and maintenance of schistosome life cycles. In *Proc. First Sino-Am. Sym.* Vol. 1, 34–48, (1987). https://www.scienceopen.com/document?vid=1dee493b-bed4-4858-993a-3e632d273a0f

[52] Shaabani E, Amini S, Kharrazi S, Tajerian R (2017). Curcumin coated gold nanoparticles: Synthesis, characterization, cytotoxicity, antioxidant activity and its comparison with citrate coated gold nanoparticles. Nanomed. J..

[53] Larasati YA (2018). Curcumin targets multiple enzymes involved in the ROS metabolic pathway to suppress tumor cell growth. Sci. Rep..

[54] Panic G, Ruf MT, Keiser J (2017). Immunohistochemical investigations of treatment with Ro 13–3978, praziquantel, oxamniquine, and mefloquine in *Schistosoma mansoni*-infected mice. Antimicrob. Agents Chemother..

[55] Cowan N, Keiser J (2015). Repurposing of anticancer drugs: In vitro and in vivo activities against *Schistosoma mansoni*. Parasit. Vectors..

[56] Jusnita N, Haditjaroko L, Yusron M, Noor E (2014). Production of nanocurcumin from Curcuma Xanthorriza Roxb. by homogenization. J. Biol. Agric. Health..

[57] Duvall RH, De Witt WB (1967). An improved perfusion technique for recovering adult schistosomes from laboratory animals. Am. J. Trop. Med. Hyg..

[58] Ali M, Eldahab MA, Mansour HA, Nigm A (2016). Schistosoma mansoni: Antiparasitic effects of orally administered *Nigella sativa* oil and/or *Chroococcus turgidus* extract. Acta Biol. Hung..

[59] Lombardo FC (2019). Life cycle maintenance and drug-sensitivity assays for early drug discovery in *Schistosoma mansoni*. Nat. Protoc..

[60] Yepes E (2015). Inhibition of granulomatous inflammation and prophylactic treatment of schistosomiasis with a combination of edelfosine and praziquantel. PLoS Neglect. Trop. D.

[61] Harris HR (1900). On the rapid conversion of haematoxylin into haematein in staining reactions. J. Appl. Microsc..

[62] Hussein A, Rashed S, El Hayawan I, El-Sayed R, Ali H (2017). Evaluation of the anti-schistosomal effects of turmeric (*Curcuma longa*) versus praziquantel in *Schistosoma mansoni* infected mice. Iran. J. Parasitol..

